# Pan-Piscine Orthoreovirus (PRV) Detection Using Reverse Transcription Quantitative PCR

**DOI:** 10.3390/pathogens10121548

**Published:** 2021-11-27

**Authors:** Julie Zhao, Niccolò Vendramin, Argelia Cuenca, Mark Polinski, Laura M. Hawley, Kyle A. Garver

**Affiliations:** 1 Pacific Biological Station, Department of Fisheries and Oceans, Nanaimo, BC V9T 6N7, Canada; julieyzhao@hotmail.com (J.Z.); Mark.Polinski1@usda.gov (M.P.); Laura.Hawley@dfo-mpo.gc.ca (L.M.H.); 2 Unit for Fish and Shellfish Diseases, National Institute of Aquatic Resources, Technical University of Denmark, 2800 Lyngby-Taarbæk, Denmark; niven@aqua.dtu.dk (N.V.); arcun@aqua.dtu.dk (A.C.)

**Keywords:** piscine orthoreovirus (PRV), real time PCR, genotypes, validation, sensitivity, specificity, reproducibility

## Abstract

Piscine orthoreovirus (PRV) infects farmed and wild salmon and trout species in North America, South America, Europe, and East Asia. PRV groups into three distinct genotypes (PRV-1, PRV-2, and PRV-3) that can vary in distribution, host specificity, and/or disease potential. Detection of the virus is currently restricted to genotype specific assays such that surveillance programs require the use of three assays to ensure universal detection of PRV. Consequently, herein, we developed, optimized, and validated a real-time reverse transcription quantitative PCR assay (RT-qPCR) that can detect all known PRV genotypes with high sensitivity and specificity. Targeting a conserved region at the 5′ terminus of the M2 segment, the pan-PRV assay reliably detected all PRV genotypes with as few as five copies of RNA. The assay exclusively amplifies PRV and does not cross-react with other salmonid viruses or salmonid host genomes and can be performed as either a one- or two-step RT-qPCR. The assay is highly reproducible and robust, showing 100% agreement in test results from an inter-laboratory comparison between two laboratories in two countries. Overall, as the assay provides a single test to achieve highly sensitive pan-specific PRV detection, it is suitable for research, diagnostic, and surveillance purposes.

## 1. Introduction

Piscine orthoreovirus (PRV) is a segmented double-stranded RNA virus in the family *Reoviridae*, genus *Orthoreovirus*, that predominately infects salmonids [1]. The PRV genome consists of 10 segments with three long (L1, L2, and L3), three medium (M1, M2, and M3), and four short segments (S1, S2, S3, and S4) [2,3]. Phylogenetic analyses have differentiated three main PRV genotypes, denoted PRV-1, PRV-2, and PRV-3, [4,5], each of which have, under some circumstances, demonstrated the capacity to cause circulatory disease in farmed salmonids. PRV-1, the genotype most well studied, includes strains demonstrated to cause heart and skeletal muscle inflammation (HSMI) in Atlantic salmon (*Salmo salar*) [6,7]. PRV-2 has been shown to cause anemia in coho salmon (*Oncorhynchus kisutch*), and experimental infections of PRV-3 have generated heart inflammation in rainbow trout (*Oncorhynchus mykiss)* [5,8].

PRV diseases have been aquaculture associated, but concerns have been raised regarding the risk of PRV to natural salmon and trout populations. Surveillance studies reveal widespread distribution with PRV detections recorded in North America, South America, Europe, and Asia [5,9,10,11,12,13,14,15] and are typically observed at higher prevalence in farmed fish. However, a significant limitation to understanding the worldwide distribution of PRV has stemmed from the inability to universally detect all known types of PRV using one broad-based diagnostic method. Consequently, PRV surveillance and monitoring efforts to date have employed genotype-specific assays, thereby imposing a bias in that the reported detections are restricted to the genotype for which they were specifically tested. For instance, in North America, PRV-1 is ubiquitous in farmed salmon populations, while PRV-2 and PRV-3 have not been reported; however, few surveillance programs have specifically looked for these other genotypes. Additionally, certain PRV strains not associated with disease states [16,17] are not prioritized for genotype-specific screening and may be under-represented in fish populations.

As PRV has not been reliably cultured in vitro [18], the use of cell culture as a broad based detection method has remained unsuccessful. Hence, detection methods for PRV have largely been molecular based, and, given the sequence divergence between genotypes, assays targeting PRV-1 [3] proved non-reactive with PRV-2 or PRV-3; similarly, PCR assays designed to detect PRV-2 [5] and PRV-3 [19] are neither cross-reactive to each other nor to PRV-1. Although instrumental in the genotype specific detection of PRV, these assays are problematic for surveillance particularly in regions where the occurrence of PRV is unknown or where multiple genotypes have been observed to co-circulate, such as PRV-1 and PRV-3 in Norway, Denmark, Germany, France, the United Kingdom, and Chile [2,3,4,9,10,15,20,21,22,23,24]. Thus, a pan-PRV assay capable of detecting all known genotypes would be beneficial to fully understand the distribution and co-occurrence of the PRV genotypes.

To this end, we developed a simple, highly reproducible, real-time quantitative PCR for the universal detection of all known PRV genotypes. This pan-PRV assay has been validated across two laboratories and can be utilized as either a one-step or two-step assay to detect PRV-1, PRV-2, and PRV-3 RNA sequences with high efficiency and specificity with no cross-reactivity with host genomes or common salmonid viruses. Overall, the pan-PRV assay is highly sensitive, specific, repeatable, and reproducible, and it is suitable for use in a diagnostic setting.

## 2. Results

### 2.1. Pan-PRV RT-qPCR Assay Development and Optimization

#### 2.1.1. Primer and Probe Design 

Alignment of six PRV reference genomes revealed two areas of sequence conservation within each of the M2 and S1 segments (Figure S1). As the 5′ end of the M2 segment between bases 1–131 (numbering based on GenBank Accession KY429947) contained the greatest area of conservation, three primer and probe sets were designed to complement this region (Figure S1) with degenerate bases incorporated into the primer sets in instances where nucleotide heterogeneity occurred across PRV genotypes (Table S1).

#### 2.1.2. Primer and Probe Set Elimination and qPCR Optimization

**Figure 1:**
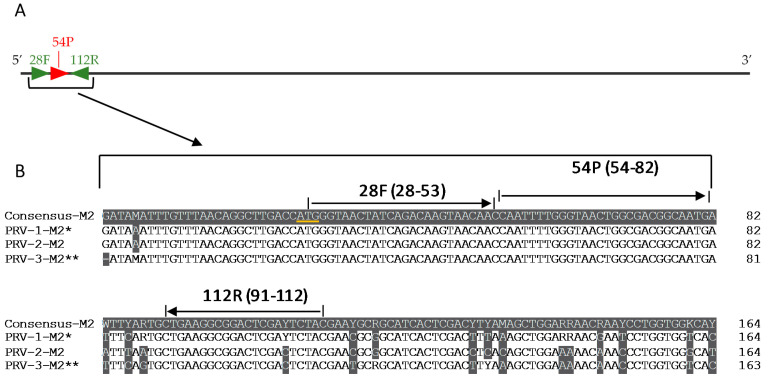
(**A**). The PRV M2 segment with set 1 primers and probe (28F, 54P, 112R). Genome base numbers according to Norwegian PRV-1b isolate NOR2012_V3621 (accession number KY429947). (**B**). Partial nucleotide alignment showing the PRV-1, PRV-2, and PRV-3 M2 segments with the corresponding consensus sequences. The start codon is underlined in yellow, and the locations of the primers (28F and 112R) and probe (54P) are marked with arrows. The PRV-1-M2* sequence is a consensus of 37 PRV-1 isolates. The PRV-3-M2** is a consensus sequence compiled from six PRV-3 isolates.

Three primer and probe sets targeting the M2 segment were initially assessed for their ability to amplify a dilution series of genotype PRV-1 isolate 16-005. As SYBR green assays, each of the three primer sets generated a prominent PRV specific PCR product identified as a single peak upon melting curve analysis (data not shown). As TaqMan assays, primer and probe set 1 (Figure 1, Table 1) recorded the highest amplification efficiency (95.87%, slope: −3.43) among the three primer/probe sets and was selected for further optimization (Table S2). Primer concentration, probe concentration, and annealing temperature were assessed independently, and the optimal reaction conditions for primer/probe set 1 were determined to be 600 nM for each primer, 200 nM probe, and an annealing temperature of 60 °C (Table S3). These reaction conditions were hereafter employed in defining the performance characteristics of the optimized two-step pan-PRV assay. 

### 2.2. Pan-PRV RT-qPCR Assay Universality, Exclusivity, and Sensitivity

#### 2.2.1. Universal Detection of PRV Genogroups 

The pan-PRV assay detected all known PRV genotypes and sub-genotypes at efficiencies greater than 88% (Table S4). Furthermore, in silico analysis of primer and probe set 1 to an expanded set of publicly available PRV M2 sequences (Table S5) confirmed sequence conservation across the 5′ end region targeted by the primer and probe. Within this region, only one heterogenic nucleotide within the reverse primer sequence was observed across PRV genotypes and existed as either a cytosine or uracil (Figure S2). Hence, incorporation of a degenerate base at this position within the reverse primer permitted complementarity regardless of PRV genotype. Additionally, internal placement of this degenerate base within the reverse primer facilitated tolerance to other potential mismatches should they occur at this heterogenic site. The pan-PRV assay conducted with a modified reverse primer (112R-Y) incorporating an uncomplimentary base at this position (Table S1) revealed nearly equivalent qPCR efficiency (87.12%) as performed with the proper set 1 reverse primer (88.63%).

#### 2.2.2. Exclusivity of Pan-PRV RT-qPCR 

Amplification of the pan-PRV assay was exclusive to PRV. Experimentally, the pan-PRV assay did not amplify other salmon and trout viruses, which included salmonid alphavirus (SAV), infectious pancreatic necrosis virus (IPNV), infectious salmon anemia virus (ISAV), infectious hematopoietic necrosis virus (IHNV), viral hemorrhagic septicemia virus (VHSV), aquareovirus A, and chum salmon reovirus (CSRV) (Table S6). Additionally, the pan-PRV assay did not cross-react with nucleic acid samples (RNA and DNA) extracted from Atlantic salmon, chinook salmon, sockeye salmon, pink salmon, and chum salmon (Table S6), nor were any significant sequence complementarities identified between set 1 primer/probe sequences and publicly available salmon and trout reference genomes (data not shown). Furthermore, in silico experiments failed to identify any sequence homology between primer/probe set 1 sequences and related fish reoviruses. Specifically, the large-mouth bass reovirus (LMBRV), Etheostoma fonticola aquareovirus (EFA), chum salmon reovirus (CSRV), golden shiner reovirus (GSRV), American grass carp reovirus (AGCRV), fall chinook aquareovirus (FCA), and green river chinook virus (GRCV) (accession numbers listed in Supplementary Materials Table S7) did not reveal any significant conservation to the PRV M2 sequence that primer/probe set 1 target (Figure S3). 

#### 2.2.3. Pan-PRV qPCR Sensitivity

**Figure 2:**
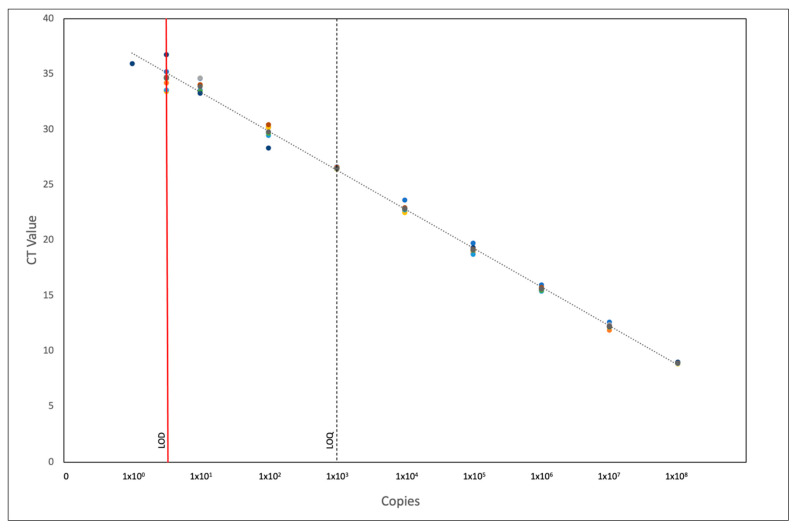
Pan-PRV qPCR APC standard curve showing Ct values for all 8 replicates ranging from 10^8^ copies to 1 copy per reaction. Limit of detection (LOD) and limit of quantification (LOQ) are labelled using solid red and dotted black lines respectively.

The amplification efficiency of PRV-1 isolate 16-005ND and an artificial positive control (APC) were 95.06% (slope: −3.44) and 90.44% (slope: −3.57), respectively, across a dynamic range of 10^6^ to 100 copies per reaction. Given the similar qPCR reaction efficiencies, the APC was used to determine the limit of detection (LOD) and limit of quantification (LOQ) of the pan-PRV assay. The assay showed reliable amplification from 10^8^ to 5 copies per reaction, as 8/8 replicates were detected across all dilutions, while a final dilution of one copy was only detected in one of eight replicates, establishing five copies as the LOD for the pan-PRV assay (Figure S4). The intra-assay coefficient of variance (CV) across all dilutions ranged from 3.78–59.96%, with the lower copy numbers generally showing greater variance between replicates. The % of CV calculated across eight replicates and over six APC concentrations (10^8^ to 10^3^ copies) was within the recommended threshold of <25% [25]. The limit of accurate quantification, the lowest number of template copies that the assay could quantify with a less than 25% coefficient of variance (CV), was 1000 copies per reaction (Figure 2).

### 2.3. One-Step Pan-PRV RT-qPCR

Performed as a one-step assay, the pan-PRV RT-qPCR achieved analytical parameters comparable to the two-step assay. Optimized using 400 nM primers and 200 nM probe, the one-step assay proved to be 100% efficient over a 9-point 10-fold serial dilution of APC template and reliably detected 10 copies per reaction (Figure S5). Further the one-step assay provides universal PRV detection, recognizing all three genogroups of PRV with similar Ct values to those obtained using genogroup-specific assays (Table S8).

### 2.4. Reproducibility and Robustness—Inter-Laboratory Comparison 

#### Inter-Laboratory Proficiency Test

**Figure 3:**
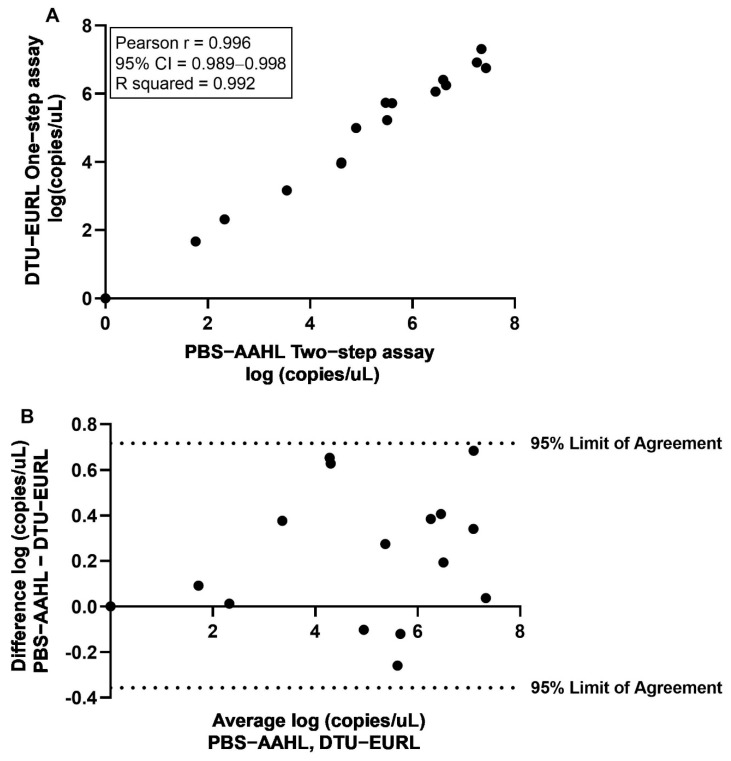
Comparison of PRV viral load in inter-laboratory proficiency panel samples processed at European Union Reference Laboratory for fish and crustacean diseases at the Danish Technical University (DTU-EURL) using the pan-PRV one-step assay vs. the two-step assay employed at the Pacific Biological Station Aquatic Animal Health Laboratory (PBS-AAHL). (**A**). Pearson’s correlation plot and correlation coefficient of log (copies/µL). (**B**). Bland–Altman plot comparing log copies/μL determined using two-step assay (PBS-AAHL) vs. one-step assay (DTU-EURL).

The pan-PRV assay proved to be reproducible and robust, as there was 100% agreement of results between two laboratories, despite significant differences in reagents, equipment, and cycling conditions (i.e., one-step vs. two-step reaction). Each laboratory correctly identified all PRV-positive and -negative samples within an inter-laboratory proficiency panel (Table S9). Additionally, quantification of viral load in the samples showed exceptionally high concordance between laboratories with a nearly perfect positive correlation coefficient (Figure 3A). Further, a Bland–Altman plot comparing the log of the viral load for the two-step method versus a one-step method did not reveal any significant bias between methods, and all data points fell within the 95% limits of agreement ranging from −0.3568 to 0.7163 (Figure 3B). 

## 3. Discussion

Here, we report the development of an RT-qPCR assay for the pan-specific detection of PRV RNA for either surveillance, research, or diagnostic purposes. To date, PRV detection assays have been constrained to specific genotypes, while the pan-PRV assay developed herein proved to have high analytical sensitivity reliably detecting as few as five copies per reaction regardless of the PRV genotype. The pan-PRV assay yielded highly repeatable results, both within and between assay runs, and is highly reproducible between laboratories when performed as either a one- or two-step assay. Furthermore, the assay was highly specific to the detection of PRV and did not cross-react with related fish reoviruses, common salmonid viruses, or salmonid genomes. With the capacity to detect all PRV types, the pan-PRV assay represents a broad-based screening tool that can be used to better characterize the host, geographic, and phylogenetic range of this virus.

Previously reported genotype-specific PRV assays have targeted sequences of either the L1, L2, or S1 segments [3,5,19]; however, the pan-PRV assay designed herein targets an 85-base sequence within the 5′ terminus of the M2 segment. While other regions of the M2 segment have high diversity and have been important in characterizing PRV phylogeny [3,5,6,19,26], the 5′ end region remains highly conserved across the genotypes. This region of the gene codes for the N-terminus of the µ1 outer capsid protein predicted to mediate cell membrane disruption [2,27] and, in particular, includes a myristoylation site (at G_2_) observed across homologous reovirus proteins, such as mammalian reovirus (MRV) µ1 protein and avian orthoreovirus (ARV) µB protein [28,29,30,31]. As the N-terminal *N*-myristoylated fragment μ1N is thought to be critical for penetration of the host cell membrane during cell entry, functional conservation is likely necessary and may, in part, constrain sequence divergence of this gene region, thereby enhancing its suitability as a universal target for detection of PRV, including not only those genotypes previously identified but also potentially unknown PRV genotypes. 

In addition, the M2 segment is a reliable indicator of the presence of PRV as a result of its stable expression regardless of the PRV phase of infection. PRV infection is typically characterized by distinct phases (as reviewed in 1) that are delineated by an entry and dissemination period that leads to peak systemic replication, followed by viral persistence and, in some instances, clearance. While the ssRNA component of all ten PRV segments has been detected during infection, significant proportional variation in the quantity of particular transcripts has been documented and is dependent upon the phase of infection. Time-course sampling of PRV-1-infected Atlantic salmon demonstrated that the M2 segment maintained relatively stable proportional ssRNA expression across the early, peak, and persistent phases of infection, while expression of other segments was variable [17,32]. Importantly, despite substantial decreases in PRV load during persistent phase infections, M2 RNA was readily detected from infected fish; in fact, the µ1 protein (encoded by M2) was the only PRV protein that could be detected after the peak of virus protein production, suggesting a possible role of this segment in persistent infection [32]. Thus for diagnostic purposes, the M2 segment provides an ideal target to facilitate low-level PRV infections.

For diagnostic assays to gain widespread use, it is essential that they are not only sensitive and specific but also are robust (i.e., perform well under different operating conditions) and reproducible across laboratories. To this end, we validated the pan-PRV assay to be utilized as either a one- or two-step RT-qPCR. Combining the cDNA and qPCR reactions into a single tube, the one-step assay is less labor intensive and generally faster, while the two-step assay, which uses random hexamers to generate cDNA, affords the laboratory the option of archiving cDNA that can be screened for other RNA viruses and/or used for confirmatory testing. Regardless of what method is preferred by a laboratory, the pan-PRV assay proved equally sensitive and specific when employed as either a one- or two-step assay, suggesting the pan-PRV assay to be relatively robust to operating conditions. Furthermore, through inter-laboratory comparison testing, the pan-PRV assay was highly reproducible between two laboratories, achieving 100% agreement in test results. 

Given the potential for co-circulating genotypes across the geographic range of PRV, it is imperative for PRV surveillance and monitoring programs to employ screening tests that are capable of detecting PRV irrespective of its genotype. Employed as a single broad-based screening test, the pan-PRV assay provides the universal detection of PRV, thereby eliminating the need to utilized multiple, independent, genotype-specific assays to ascertain the presence of PRV. In the event positive amplification is observed with the pan-PRV assay, genotype can be ascribed through sequencing or genotype-specific assays.

## 4. Materials and Methods

### 4.1. Pan-PRV RT-qPCR Assay Development and Optimization

#### 4.1.1. PRV Source Material, RNA Extraction, and cDNA Synthesis

Blood and tissue samples infected with PRV strains representative of the known PRV genotypes and sub-types (Table 2) were used in the development and validation of the pan-PRV assay. Total RNA was extracted from all samples using TRIzol^®^ Reagent (Ambion) according to manufacturer’s instructions. Samples were homogenized with a TissueLyser (Qiagen) for 2 min at 25 Hz using 5 mm stainless steel beads. PRV-positive RNA extracts were stored at −80 °C until they were used as template for cDNA synthesis, where approximately 1 µg total RNA was denatured at 95 °C for 5 min, cooled to 4 °C, and reverse transcribed using the High-Capacity cDNA Reverse Transcription Kit (Life Technologies) according to the manufacturer’s instructions. The cDNA products were stored at −20 °C. 

#### 4.1.2. Primer and Probe Design 

Six full genome sequences (Table S10), reflecting the known genetic diversity of PRV, were aligned using the Geneious Prime 2020.1.2 software platform to create a consensus sequence. The M2 segment was selected and targeted for primer and probe design based on sequence homology across all genotypes. Three primer/probe sets were chosen, and degenerate bases were incorporated, where nucleotides varied across genotypes. Probes contained 5′ fluorescently labeled 6-carboxyfluorescein reporter dye (FAM) and 3′ minor groove binding (MGB) quencher (ThermoFisher, Waltham, MA, USA). 

#### 4.1.3. Primer and Probe Set Elimination and qPCR Optimization

To determine the suitability of the three primer and probe sets to amplify PRV, each set was assessed for their capacity to amplify PRV-1a isolate 16-005 over a 3-point 100-fold dilution series. RNA was extracted, and cDNA was synthesized as described above. For initial assessment, qPCR reactions (15 µL) contained 1 µL cDNA (~50 ng RNA), 7.5 µL 2X TaqMan Universal PCR Master mix (Thermofisher), and a primer/probe set with primers fixed at a concentration of 400 nM and probe at concentration of 300 nM. The reactions were run on a StepOnePlus™ real-time PCR system (Applied Biosystems) with the qPCR data analyzed using the StepOnePlus™ Software v2.3 and thresholds set to 0.02ΔRn. As a positive control and comparator, a primer and probe specific to PRV-1 [3] was performed in parallel with all reactions undergoing cycling parameters as described in Polinski et al. (2019). The primer and probe set with the highest qPCR efficiency was selected for further optimization. Primers were optimized using Power SYBR™ green (Applied Biosystems), a PRV-1a (16-005) 5-fold dilution series as template, and forward and reverse primer concentrations ranging from 200 nM to 800 nM in 200 nM increments. Thermocycling conditions included an initial hold at 95 °C for 10 min; 40 cycles of 95 °C for 10 s; 60 °C for 30 s; and a melt curve of 95 °C for 15 s, 55 °C for 60 s, and 95 °C for 15 s. After the optimal primer concentration was determined, the probe was assessed at concentrations ranging from 200 nM to 400 nM in 100 nM increments. TaqMan cycling parameters consisted of an initial hold at 95 °C for 10 min, 40 cycles of 95 °C for 10 s, and 60 °C for 20 s. Thresholds for all TaqMan assays were manually set to 0.02ΔRn. Lastly, the annealing temperatures of 55, 58, 60, 62, and 65 °C were evaluated. All subsequent tests to define the performance characteristics of the pan-PRV assay were performed utilizing the optimal primer and probe concentrations and associated cycling conditions. 

### 4.2. Pan-PRV RT-qPCR Assay Universality, Exclusivity, and Sensitivity

#### 4.2.1. Universal Detection of PRV Genotypes

To assess the capacity of primer and probe set 1 to universally detect PRV, six PRV isolates representing all known genotypes of PRV (Table 2) were used as template in the pan-PRV qPCR assay. Amplification was assessed over an 8-point 10-fold dilution series of each template, and reaction efficiencies were evaluated. In silico analysis was also performed by aligning primer and probe set 1 with forty-four publicly available PRV M2 sequences in Geneious Prime.

#### 4.2.2. Exclusivity of Pan-PRV RT-qPCR 

Using nucleotide BLAST, sequence similarities of set 1 primer and probe sequences were first evaluated across all members of Reoviridae (taxid: 10880), excluding the piscine orthoreovirus (taxid: 1157337), piscine orthoreovirus 2 (taxid: 1828353), and piscine orthoreovirus 3 (taxid: 2153377). Results were assessed for E-values < 5. Refined sequence alignments of primer and probe set 1 with specific fish reoviruses, including LMBRV, EFA, CSRV, GSRV, AGCRV, FCA, and GRCV, were performed in Geneious Prime. Corresponding accession numbers are listed in Table S7. Experimentally, the pan-PRV RT-qPCR was tested for cross-reactivity using 1 μL of the following cell culture amplified salmonid viruses in a 15 μL reaction volume: SAV, IPNV, IHNV, VHSV, ISAV, and CSRV. To assess potential off-target binding of set 1 primers/probe to salmonid host genomes, nucleotide BLAST (blastn) queries were conducted using default parameters for short input sequences. Queries were run against all available nucleotide sequences for salmonids (taxid: 8015), salmons and trouts (taxid: 8006), pink salmon (taxid: 8017), chum salmon (taxid: 8018), coho salmon (taxid: 8019), sockeye salmon (taxid: 8023), chinook salmon (taxid: 74940), Atlantic salmon (taxid: 8030), masu salmon (taxid: 8020), rainbow trout (taxid: 8022), river trout (taxid: 8032), lake trout (taxid: 8040), and cutthroat trout (taxid: 30962). Specific reference genomes were also analyzed with blastn using the same parameters (Table S11). Results were assessed and deemed significant if all three primer/probe sequences produced E-values < 5 on one genome. Empirically, the pan-PRV RT-qPCR was screened for cross-reactivity with five salmon species. Nucleic acids were extracted from Atlantic salmon, chinook salmon, sockeye salmon, and pink salmon kidneys and chum salmon fin tissue using a DNeasy Blood and Tissue Kit according to manufacturer’s instructions (Qiagen). The five extracted nucleic acid sample were each screened in duplicate using 1 μL of sample in a 15 μL reaction for the pan-PRV RT-qPCR assay. 

#### 4.2.3. Pan-PRV qPCR Sensitivity

To determine the limit of detection of the pan-PRV assay, a 10-fold dilution series of artificial positive control (APC) (Integrated DNA Technologies; Figure S6) ranging from 10^8^ to 1 copies per qPCR reaction was run across 8 replicates of each dilution. To evaluate whether the APC is a suitable proxy for absolute quantification of PRV-infected tissues, reaction efficiencies calculated from APC standards were compared with efficiencies determined from standards curves generated from an 8-point 10-fold dilution series of PRV-1a RNA. The pan-PRV assay limit of detection was defined as the last dilution point where 100% of replicates yielded a Ct value, while the limit of accurate quantification was defined as the last dilution where the coefficient of variation between replicates was less than 25%.

### 4.3. One-Step Pan-PRV RT-qPCR

A one-step RT-qPCR assay utilizing the pan-PRV set 1 primers and probe was also developed. Briefly, 28 μL reactions containing 1x TaqPath™ 1-Step RT-qPCR Master Mix, 400 nM of each primer, 200 nM probe, and 5 μL RNA template were run on a Stratagene MxPro with the following cycling conditions: 25 °C for 2 min, 53 °C for 15 min, 95 °C for 5 min, 45 cycles of 95 °C for 15 s, 60 °C for 1 min. The limit of detection was determined using a 10-fold dilution series of APC ranging from 10^9^ to 1 copy per reaction. The one-step pan-PRV RT-qPCR described here was also compared to previously published genotype specific RT-qPCR assays using a panel of 15 archived samples representing PRV genotypes 1–3. One-step RT-qPCR targeting PRV-1 is described by Palacios et al. (2010), two-step RT-qPCR assay targeting PRV-2 is described by Takano et al. (2016), and one-step RT-qPCR targeting PRV-3 is described by Olsen et al. (2015).

### 4.4. Reproducibility and Robustness—Inter-Laboratory Comparison 

To investigate the robustness of the pan-PRV assay, a panel of 20 blinded samples containing low, medium, and high viral load of PRV-1, PRV-2, PRV-3a, PRV-3b, or no PRV target was processed by two laboratories: the Aquatic Animal Health Laboratory at the Pacific Biological Station (PBS-AAHL) and the European Union Reference Laboratory for fish and crustacean diseases at the Danish Technical University (DTU-EURL). The PBS-AAHL processed the panel using the two-step pan-PRV assay, while the DTU-EURL utilized the one-step pan-PRV assay described above. Each laboratory included an APC standard curve (10^7^ to 10 copies per reaction) and reported absolute copy number (copies/ μL RNA) as well as mean Ct values for each sample. The data provided were transformed into log scale and analyzed using GraphPad Prism to determine Pearson correlation coefficient and to generate correlation and Bland–Altman plots. 

## 5. Conclusions 

The optimized pan-PRV RT-qPCR assay described here provides universal detection of all known PRV genotypes. Performed as either a one- or two-step assay, the pan-PRV assay is highly sensitivity and specific to PRV while excluding cross-reaction with other common salmonid viruses and salmonid host genomes. Being robust and reproducible, the pan-RPV assay is suitable for research, diagnostic, and surveillance purposes.

## Figures and Tables

**Table 1:** Set 1 primer and probe sequences and associated parameters designed for universal detection of the M2 segment of PRV-1, PRV-2, and PRV-3. Degenerate bases are in bold and underlined. Tm: melting temperature, FAM: 6-carboxy fluorescein, MGBNFQ: minor groove binding non-fluorescent quencher.

Target	Name	Nucleotide Sequence (5′→3′)	Tm (°C)	GC (%)	Amplicon (bp)
PRV M2	28F	TGGGTAACTATCAGACAAGTAACAAC	58.8	39	
	112R	GTAGA**R**TCGAGTCCGCCTTCAG	60.5–62.1	57	85
	54P	FAM-CAATTTTGGGTAACTGGCGACGGCAATGA-MGBNFQ	68.2	48	

**Table 2:** PRV-1, PRV-2, and PRV-3 isolates used as reference materials for assay validation.

Isolate.	Genotype	GenBank Accession No.	Source		Reference
			Host Species	Tissue	
16-005	PRV-1a	MH347359-MH347368	Atlantic salmon	Blood	[17]
r17_1227	PRV-1a	R17_1227: (MW354796, MW354808, MW354820, MW354832, MW354844, MW354856, MW354868, MW354880, MW354892, MW354904)	Atlantic salmon	Blood	[33]
NOR2012_V3621	PRV-1b	KY429943-KY429952	Atlantic salmon	Blood	[6]
---	PRV-2	LC145608-LC145617	Coho salmon	Heart	[5]
NOR/060214	PRV-3a	MG253807-MG253816	Rainbow trout	Spleen and heart (pooled)	[4]
DK/PRV315	PRV-3b	MW012855-MW012864	Rainbow trout	Spleen and heart (pooled)	[14]

## Data Availability

Not applicable.

## Supplementary Materials

The following are available online at https://www.mdpi.com/article/10.3390/pathogens10121548/s1. Table S1: Primer and probe sequences targeting the 5′ end of the M2 segment of piscine orthoreovirus (PRV) 1, 2, and 3. Degenerate bases are in bold and underlined. Tm: melting temperature, FAM: 6-carboxy fluorescein, NGBNGQ: minor groove binding non-fluorescent quencher. Table S2: Amplification efficiencies for detection of a PRV-1 sample using the pan-PRV primer/probe sets 1, 2, and 3 targeting the PRV M2 segment compared to a previously established PRV-1 genotype specific assay targeting the L1 segment. Table S3: Determination of optimal primer and probe concentrations for the pan-PRV set 1 primers/probe through screening of a PRV-1 isolate. Optimal primer and probe concentrations are highlighted in bold. Table S4: Pan-PRV RT-qPCR amplification efficiencies for all known PRV genotypes. Table S5: List of 44 PRV isolate M2 segments used for the in silico analysis of primer/probe set 1. Table S6: Pan-PRV RT-qPCR exclusivity test using various salmon viruses, trout viruses, and naive salmon nucleic acids. A Ct value of “Undetermined” corresponds to absence of target amplification. Table S7: Accession numbers for fish reoviruses assessed for cross-reactivity with pan-PRV set 1 primers/probe. Table S8: Comparison of PRV detection in tissue samples using pan-PRV one-step RT-qPCR assay and genotype specific assays. Table S9: Inter-laboratory proficiency panel results from the Aquatic Animal Health Laboratory at the Pacific Biological Station (PBS-AAHL) and the European Union Reference Laboratory for fish and crustacean diseases at the Danish Technical University (DTU-EURL). Table S10: PRV reference genomes used for primer and probe design of the pan-PRV RT-qPCR assay. Table S11: Salmon and trout host genomes used for in silico analysis of pan-PRV set 1 primer/probe exclusivity assessment. Figure S1: Geneious alignments illustrating the conserved regions across six PRV genomes. A. Consensus sequence and conserved areas within M2 segment. Pan-PRV primer/probe sets targeting the conserved region at the 5′ end of the M2 segment are identified. B. Consensus and conserved region within S1 segment. Figure S2: Alignment of 44 PRV M2 segments. Pan-PRV set 1 primer and probe binding sites identified on the consensus sequence. Only the pan-PRV reverse primer contained a degenerate base to address the heterogenic site at base 107. Figure S3: PRV M2 nucleotide sequence aligned with homologous gene segments from seven related reoviruses. Pan-PRV primer and probe sequences do not share any significant sequence homology with related reovirus. Figure S4: Limit of detection (LOD) and limit of quantification (LOQ) calculations determined for pan-PRV assay using an artificial positive control (APC) as template. Figure S5: Pan-PRV one-step RT-qPCR standard curve and amplification plot from a 9-point serial 10-fold dilution from 10^9^ to 1 copy resulting in an efficiency of 100.4% and an R^2^ of 1.0. Figure S6: Sequence for artificial positive control fragment containing PRV M2 segment targeted by pan-PRV set 1 primers and probe. Forward primer (28F) in bold, reverse primer (112R) in bold and underlined, and probe (54P) underlined.
